# Effect of β-Lactamase inhibitors on in vitro activity of β-Lactam antibiotics against *Burkholderia cepacia* complex species

**DOI:** 10.1186/s13756-016-0142-3

**Published:** 2016-11-16

**Authors:** Annelien Everaert, Tom Coenye

**Affiliations:** Laboratory of Pharmaceutical Microbiology, Ghent University, Ottergemsesteenweg 460, 9000 Ghent, Belgium

**Keywords:** *Burkholderia cepacia* complex, Resistance, β-lactam, β-lactamase, β-lactamase inhibitors

## Abstract

**Background:**

Bacteria belonging to the *Burkholderia cepacia* complex (Bcc) are an important cause of chronic respiratory tract infections in cystic fibrosis patients. Intrinsic resistance to a wide range of antimicrobial agents, including a variety of β-lactam antibiotics, is frequently observed in Bcc strains. Resistance to β-lactams is most commonly mediated by efflux pumps, alterations in penicillin-binding proteins or the expression of β-lactamases. β-lactamase inhibitors are able to restore the in vitro activity of β-lactam molecules against a variety of Gram-negative species, but the effect of these inhibitors on the activity of β-lactam treatment against Bcc species is still poorly investigated.

**Methods:**

In the present study, the susceptibility of a panel of Bcc strains was determined towards the β-lactam antibiotics ceftazidime, meropenem, amoxicillin, cefoxitin, cefepime and aztreonam; alone or in combination with a β-lactamase inhibitor (clavulanic acid, sulbactam, tazobactam and avibactam). Consequently, β-lactamase activity was determined for active β-lactam/β-lactamase inhibitor combinations.

**Results:**

Clavulanic acid had no effect on minimum inhibitory concentrations, but addition of sulbactam, tazobactam or avibactam to ceftazidime, amoxicillin, cefoxitin, cefepime or aztreonam leads to increased susceptibility (at least 4-fold MIC-decrease) in some Bcc strains. The effect of β-lactamase inhibitors on β-lactamase activity is both strain- and/or antibiotic-dependent, and other mechanisms of β-lactam resistance (besides production of β-lactamases) appear to be important.

**Conclusions:**

Considerable differences in susceptibility of Bcc strains to β-lactam antibiotics were observed. Results obtained in the present study suggest that resistance of Bcc strains against β-lactam antibiotics is mediated by both β-lactamases and non-β-lactamase-mediated resistance mechanisms.

**Electronic supplementary material:**

The online version of this article (doi:10.1186/s13756-016-0142-3) contains supplementary material, which is available to authorized users.

## Background

Infections caused by antibiotic-resistant bacteria pose an increasing threat to public health, both in terms of human suffering and in economic terms. In addition to the costs associated with an extended hospital stay, the costs for treating infections caused by multidrug-resistant organisms are much higher than the costs for treating similar infections caused by sensitive organisms [1]. Despite the impact of these infections, the number of novel antibiotics in the pipeline is small. This is partly due to the high costs and extensive research-time associated with the development of a new antibiotic [2].

The *Burkholderia cepacia* complex (Bcc) is a group of 20 closely related opportunistic, Gram-negative pathogens [3]. Bcc species are an important cause of severe chronic respiratory infections in patients with cystic fibrosis (CF) [4]. Bcc infection in CF patients often correlates with a rapid decrease in lung function leading to a poorer prognosis, longer hospital stays and an increased risk of death. The ability of Bcc strains to form biofilms in vitro and in vivo contributes to reduced antimicrobial susceptibility, treatment failure and persistent infection [5–7]. Intrinsic resistance to a wide range of antimicrobial agents, including aminoglycosides, polymyxin, first and second generation cephalosporins, carboxypenicillins and other β-lactam antibiotics is frequently observed in Bcc strains. Resistance of Gram-negative microorganisms to β-lactam molecules is most commonly mediated by inducible or constitutively expressed β-lactamases, efflux pumps or alterations in PBPs [8, 9]. β-lactamase was first identified in 1940 as penicillinase in *Escherichia coli* even before the clinical use of penicillin, and has since been identified in a variety of other bacteria, including species of the Bcc [10–13].

β-lactam antibiotics cause cell death by inhibition of bacterial cell wall synthesis. They do so by binding to PBPs, resulting in a decreased cross-linking of peptidoglycan in the cell wall, eventually leading to cell death [8, 14]. The utility of β-lactams has become compromised through the increasing presence of both chromosomally and plasmid-encoded β-lactamase enzymes. Two classification schemes are currently used, i.e. the Ambler molecular classification (based on amino acid sequences and structural similarities) and the Bush-Jacoby-Medeiros scheme (classification according to functionality or activity against β-lactam antibiotics) [15]. In the Ambler scheme, four groups of β-lactamases are recognized; class A through D. Class A, C and D have a serine active site and class B enzymes are metallo-β-lactamases (MBLs) that need Zn^2+^ as a cofactor for their activity [10, 14]. The most commonly encountered β-lactamases are class A enzymes with TEM and SHV enzymes regularly found in Gram-negative bacteria (e.g. *E. coli* and *Klebsiella* spp.), as well as CTX-M extended-spectrum β-lactamases (ESBLs) and *Klebsiella pneumoniae* carbapenemases (KPCs) which are now also frequently encountered. Most class A enzymes can be inhibited with the commercially available β-lactamase inhibitors clavulanic acid, sulbactam or tazobactam. Class B β-lactamases (MBLs) pose a particular challenge for medicinal chemists and clinicians because thus far, none of the available inhibitors can effectively inhibit members of this class [16, 17]. Currently, treatment of MBL-producing organisms is limited to relatively toxic antibiotics (e.g. colistin) and/or antimicrobials likely to cause further development of resistance (e.g. tigecycline). EDTA, that functions as a chelator of divalent cations (including Zn^2+^), was used earlier as an active MBL inhibitor but was withdrawn from the market in 2008 due to toxicity concerns [14, 18]. Class C or AmpC β-lactamases are encoded by genes on the bacterial chromosome, although the prevalence of plasmid-mediated AmpC-enzymes is increasing. Only the novel β-lactamase inhibitor avibactam is able to inhibit class C enzymes [10, 14]. Finally, class D β-lactamases (also known as oxacillinases), are active against a broad range of β-lactam antibiotics. Clavulanic acid, sulbactam nor tazobactam inhibit these enzymes, but avibactam inhibits some class D enzymes [8, 10, 14].

The fact that some β-lactamase inhibitors restore the in vitro activity of ceftazidime against a variety of Gram-negative bacteria is known and well-established [8, 14, 19, 20], but the effect of adding these inhibitors to β-lactam antibiotics is still poorly investigated for Bcc species. Previous research on this matter led to contradictory results; Lagacé-Wiens et al. reported that avibactam does not have the ability to potentiate ceftazidime against clinical Bcc isolates, but Mushtaq et al. observed that avibactam does have variable ability to restore ceftazidime activity against Bcc isolates from patients with CF [14, 21]. These data suggest that in these Bcc isolates resistance against ceftazidime is not only mediated by expression of β-lactamases but is also due to other resistance mechanisms (efflux pumps or altered PBPs). In the present study we wanted to validate this assumptions for a larger research panel; therefore we systematically investigated the effect of β-lactamase inhibitors (clavulanic acid, sulbactam, tazobactam and the novel inhibitor avibactam) on the susceptibility of Bcc species against several β-lactam antibiotics.

## Methods

### Strains and culture conditions

The following strains were used: *B. cepacia* LMG 1222 and LMG 18821; *Burkholderia multivorans* LMG 18822, LMG 18825, LMG 13010 and LMG 17588; *Burkholderia cenocepacia* LMG 16656, LMG 18828, LMG 18829 and LMG 18830; *Burkholderia vietnamiensis* LMG 10929 and LMG 18835; *Burkholderia ambifaria* LMG 19182 and LMG 19467; *Burkholderia lata* LMG 6992 and R-9940; *Burkholderia stabilis* LMG 14294 and LMG 14086; *Burkholderia dolosa* LMG 18943 and LMG 18941; *Burkholderia anthina* LMG 20980 and LMG 20983; *Burkholderia pyrrocinia* LMG 21824; *Burkholderia ubonensis* LMG 20358 and LMG 24263; *Burkholderia latens* LMG 24064; *Burkholderia arboris* LMG 24066 and R-132; *Burkholderia seminalis* LMG 24067 and LMG 24272; *Burkholderia metallica* LMG 24068 and R-2712; and *Burkholderia contaminans* LMG 16227 and R-12710. The biological and geographic origin of every Bcc strain is presented in Additional file 1. All strains were obtained from the BCCM/LMG Bacteria Collection (Ghent, Belgium) or were kindly provided by Prof. P. Vandamme (Ghent University, Belgium). Two control strains were included; *P. aeruginosa* ATCC 27853 and *E. coli* ATCC 25922, both obtained from the ATCC collection (Manassas, VA, USA). Bacterial cultures were stored at -80 °C in Microbank vials (Prolab Diagnostics, Richmond Hill, ON, Canada) and were subcultured twice on Luria-Bertani agar (LBA; LabM Limited, Heywood, UK) before use. All cultures were incubated aerobically at 37 °C.

### Antibiotics and β-lactamase inhibitors

We used several β-lactam antibiotics of different classes including amoxicillin (AMOX; aminopenicillin), cefoxitin (CFX; 2nd generation cephalosporin), ceftazidime (CAZ; 3rd generation cephalosporin), cefepime (CFP; 4th generation cephalosporin), meropenem (MEM; carbapenem) and aztreonam (AZT; monobactam). The effect of combining these antibiotics with the β-lactamase inhibitors clavulanic acid (CLA), sulbactam (SUL), tazobactam (TAZ) and avibactam (AVI) was investigated. AMOX, CFX, CAZ, CFP, CLA, SUL and TAZ were purchased from Sigma-Aldrich (St. Louis, MO, USA). MEM was obtained from Hospira Benelux (Antwerp, Belgium), AZT from TCI Europe (Zwijndrecht, Belgium) and AVI was obtained from Adooq Bioscience (Irwin, CA, USA). The concentration range tested for CAZ and MEM was 0.25 − 128 mg/L. Higher concentrations were tested for AMOX, CFX, CFP and AZT; between 1 and 512 mg/L (according to Peeters et al. [4] and CLSI guidelines). The β-lactamase inhibitors were added at fixed concentrations as mentioned in the European Committee on Antimicrobial Susceptibility testing (EUCAST) breakpoint tables; 2 mg/L for CLA and 4 mg/L for SUL, TAZ and AVI [22].

### MIC determination

Susceptibility of the selected Bcc strains was investigated by determining minimum inhibitory concentrations (MICs) (in triplicate) of β-lactam antibiotics in the presence or absence of β-lactamase inhibitors, according to the EUCAST broth dilution guidelines using flat-bottomed 96-well microtitre plates (TPP, Trasadingen, Switzerland) [23]. Antibiotic solutions were added to the wells and two-fold dilutions were made. Planktonic cultures were grown overnight in Luria-Bertani broth (LBB; LabM, Lancashire, UK) at 37 °C. The cultures were then adjusted with double-concentrated Mueller-Hinton broth (MHB; Beckton, Dickinson & Company (BD), Erembodegem, Belgium) to obtain a final inoculum of 5 x 10^5^ cfu/ml. Plates were incubated for 24 h at 37 °C and optical density was determined at 590 nm using an Envision multilable plate reader (Perkin Elmer, Waltham, MA, USA). The MIC value is the lowest concentration of the antibiotic that completely inhibits bacterial growth [4, 23].

### β-lactamase activity assay

We explored differences in β-lactamase activity by using a β-lactamase activity assay kit (Sigma-Aldrich, St. Louis, MO, USA). This assay is based on the hydrolysis of the chromogenic molecule nitrocefin, a non-antimicrobial cephalosporin, by β-lactamase which leads to the production of a colorimetric product. Formation of this product is monitored by measuring absorbance at 490 nm in an Envision multilable plate reader; every minute, for 60 to 90 min at 25 °C. The amount of enzyme required to hydrolyze 1.0 μmol of nitrocefin per minute at pH 7.0 at 25 °C is equal to one unit of β-lactamase [10, 11, 24]. Bacterial cultures were grown in 96-well microtitre plates in the presence or absence of antibiotics. Antibiotics were added to the microtitre plates at concentrations of ¼ MIC and the β-lactamase inhibitors SUL, TAZ and AVI were added at 4 mg/L. Planktonic cultures were grown overnight in LB broth at 37 °C, then adjusted with double-concentrated MHB to obtain a final inoculum of 5 x 10^5^ cfu/ml in the 96-well plates. For every condition 10 wells in the same plate were filled and the plates were incubated at 37 °C for 24 h. After incubation, the content of the 10 wells was collected in a pre-weighed plastic tube and centrifuged at 10 000 RCF for 10 min. Then, supernatant was discarded and the pellet was weighed and resuspended with 5 μL of β-lactamase assay buffer per mg sample. Subsequently, samples were sonicated for 5 min, placed on ice for 5 min and centrifuged at 16 000 RCF at 4 °C for 20 min. 1 – 50 μL of the unknown samples was added to a clear flat 96-well plate and supplemented with nitrocefin and buffer to a final volume of 100 μL. Immediately after addition of nitrocefin, absorbance at 490 nm was measured in an Envision plate reader.

## Results and discussion

### MIC determination

For six Bcc strains (*B. cepacia* LMG 1222, *B. multivorans* LMG 18822, *B. cenocepacia* LMG 16656, *B. vietnamiensis* LMG 10929, *B. ambifaria* LMG 19182 and *B. lata* LMG 6992) and two control strains (*P. aeruginosa* ATCC 27853 and *E. coli* ATCC 25922) MIC values of all compounds and combinations were determined (Table 1). When the MIC value for a given antibiotic decreased 4-fold or more upon addition of a β-lactamase inhibitor, the inhibitor was considered to have meaningful activity. CLA showed no effect in this initial screening and was not included in further experiments. SUL, TAZ and AVI did show some effect in the initial screening and these three inhibitors were tested against a larger Bcc strain panel (Table 2).

**Table 1:**
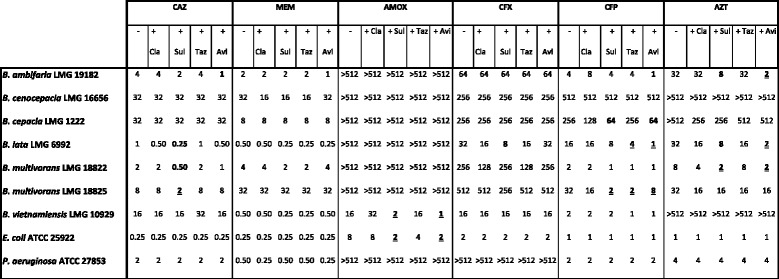
MICs of six antibiotics (in mg/L); alone or in combination with a β-lactamase inhibitor

Bold values indicate combinations leading to at least 4-fold MIC-reduction upon addition of the β-lactamase inhibitor. Underlined values indicate a shift from resistant to susceptible upon addition of the β-lactamase inhibitor, according to PK/PD (non-species related) EUCAST breakpoints. [22] “-“represents treatment with β-lactam antibiotic alone. *CAZ* ceftazidime, *MEM* meropenem, *AMOX* amoxicillin, *CFX* cefoxitin, *CFP* cefepime, *AZT* aztreonam. *Cla* clavulanic acid, *Sul* sulbactam, *Taz* tazobactam, *Avi* avibactam

**Table 2:**
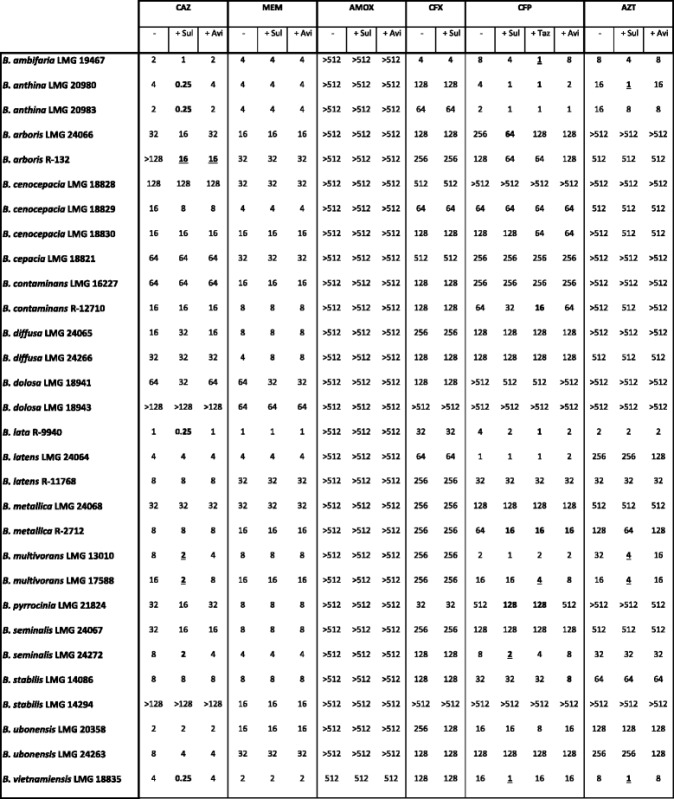
MICs of six antibiotics (in mg/L), alone or in combination with a β-lactamase inhibitor

Bold values indicate combinations leading to at least 4-fold MIC-reduction upon addition of the β-lactamase inhibitor. Underlined values indicate a shift from resistant to susceptible upon addition of the β-lactamase inhibitor, according to PK/PD (non-species related) EUCAST breakpoints. [22] “-“represents treatment with β-lactam antibiotic alone. *CAZ* ceftazidime, *MEM* meropenem, *AMOX* amoxicillin, *CFX* cefoxitin, *CFP* cefepime, *AZT* aztreonam. *Cla* clavulanic acid, *Sul* sulbactam, *Taz* tazobactam, *Avi* avibactam

In general, the MICs of each β-lactam antibiotic alone varied widely; with MIC values of CAZ ranging from 0.25 to >128 mg/L, MEM from 0.25 to 64 mg/L, AMOX from 8 to >512 mg/L, CFX from 2 to >512 mg/L and of CFP and AZT ranging from 1 to >512 mg/L. Antibiotic susceptibility results for Bcc species can be expressed as susceptible (S) or resistant (R) according to the EUCAST PK/PD (non-species related) breakpoints [22]. In vitro activity of CAZ against some Bcc strains was increased when combined with SUL or AVI; e.g. for *B. multivorans* LMG 17588 the MIC for CAZ decreased from 16 mg/L in the absence of a β-lactamase inhibitor to 2 mg/L in the presence of SUL. Hence, addition of SUL to CAZ for treatment of *B. multivorans* LMG 17588 induces a shift from R to S. Also for AMOX and AZT an increased in vitro activity against some strains was observed, in combination with SUL or AVI. However, only the addition of SUL to AZT treatment leads to a change from R to S for a slection of strains (*B. anthina* LMG 20980, *B. multivorans* LMG 13010 and *B. vietnamiensis* LMG 18835). CFP showed at least 4-fold increased activity against some Bcc strains when combined with SUL, TAZ or AVI and especially addition of TAZ and SUL leads to a shift from R to S for certain Bcc strains. Resistance towards MEM due to β-lactamases has not yet been observed in the Bcc and MEM is one of the β-lactam antibiotics with the best growth-inhibitory activity against Bcc species at clinically relevant concentrations [4, 25]. We did not observe altered susceptibility to MEM when it was combined with SUL, TAZ or AVI, confirming that Bcc strains do not produce β-lactamases that can degrade MEM. For CFX only one combination with meaningful activity was observed; MIC of CFX against *B. lata* LMG 6992 decreased from 32 mg/L in the absence of a β-lactamase inhibitor to 8 mg/L in the presence of SUL. Due to the lack of PK/PD breakpoints for CFX, it is unclear whether this change is clinically relevant. These results suggest that for most Bcc strains non-β-lactamase-mediated resistance to CFX, in the form of efflux pumps, altered PBPs, and/or reduced permeability, plays a more important role than β-lactamases in β-lactam resistance.

### β-lactamase activity assay

For the Bcc strains for which active β-lactam antibiotic/β-lactamase inhibitor combinations were identified, we investigated whether there is a relation between differences in β-lactamase activity and the altered susceptibility observed upon addition of a β-lactamase inhibitor. Therefore, a β-lactamase activity assay was used to measure β-lactamase activity. Only the combinations which showed at least a 4-fold MIC decrease upon addition of a β-lactamase inhibitor were included (i.e. CAZ + SUL, CAZ + AVI, AMOX + SUL, AMOX + AVI, CFX + SUL, CFP + SUL, CFP + TAZ, CFP + AVI, AZT + SUL and AZT + AVI).

**Fig. 1:**
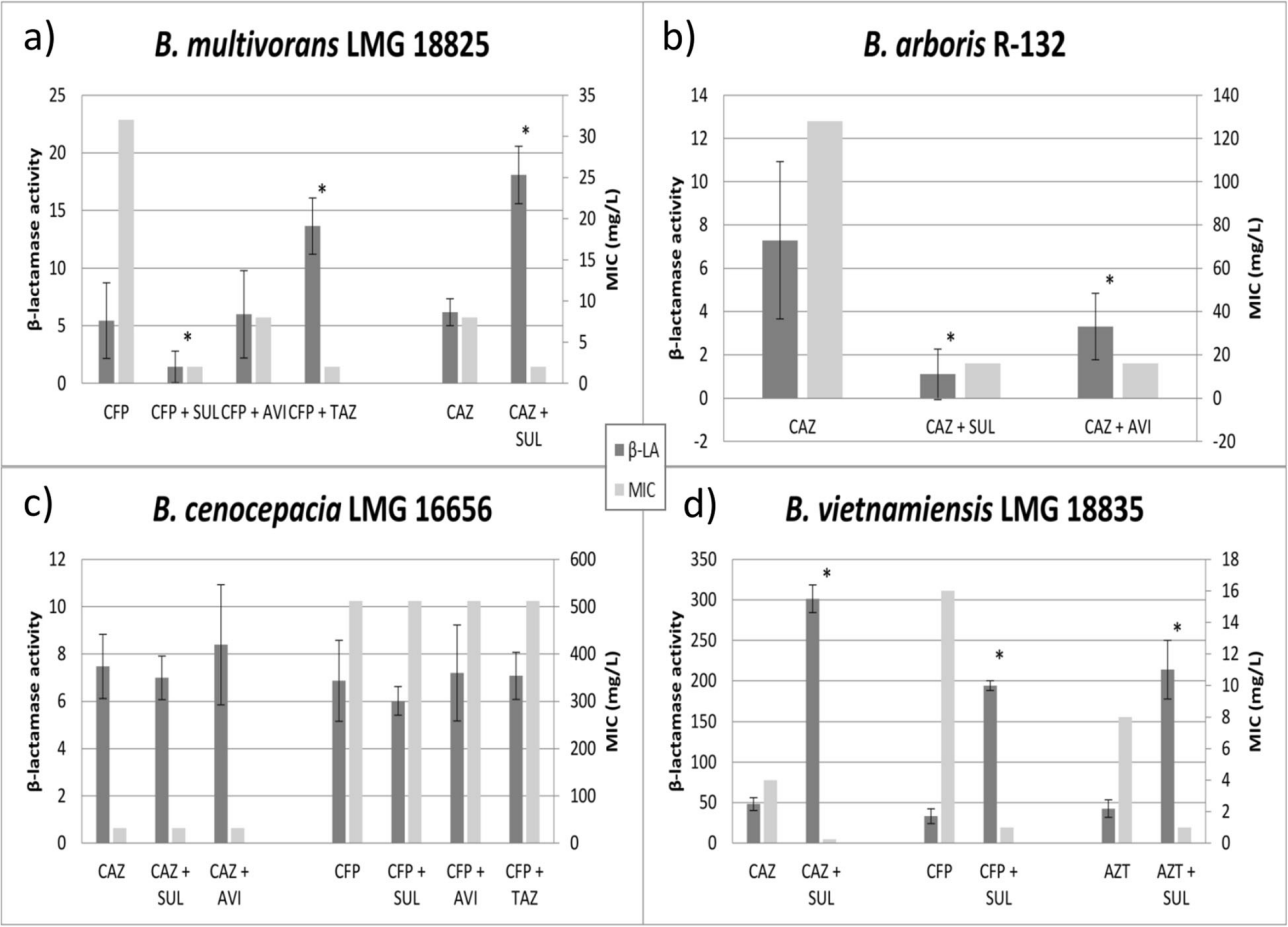
β-lactamase activity and corresponding MIC values for a selection of Bcc strains tested. Dark grey bars: β-lactamase activity relative to untreated controls, light grey bars: MIC values (mg/L) for **a**) *B. multivorans* LMG 18825, **b**) *B. arboris* R-132, **c**) *B. cenocepacia* LMG 16656 and **d**) *B. vietnamiensis* LMG 18835. * represents statistically significant differences compared to treatment with the β-lactam antibiotic alone (*p* < 0.05, *n* ≥ 3)

Differences in β-lactamase activity were explored between untreated samples, samples treated with a β-lactam antibiotic alone and samples treated with β-lactam/β-lactamase inhibitor combination treatments mentioned above (Fig. 1). Results of the β-lactamase screening are expressed as relative β-lactamase activities compared to untreated bacterial samples. For *B. multivorans* LMG 18825 (Fig. 1a) the MIC for CFP decreased from 32 mg/L in the absence of an inhibitor to 8 mg/L in the presence of AVI, or to 2 mg/L in the presence of SUL or TAZ. As expected, treatment with 0.5 mg/L CFP significantly (*p* < 0.05, *n* = 5) increased relative β-lactamase activity to 5.44 and addition of 4 mg/L SUL significantly (*p* < 0.05, *n* = 5) decreased β-lactamase activity to 1.43. However, addition of 4 mg/L AVI had no significant (p > 0.05, *n* = 5) influence on β-lactamase activity (5.99) and addition of TAZ to CFP-treatment caused a significant (*p* < 0.05, *n* = 5) increase in relative β-lactamase activity to 13.64. These data demonstrate that there is no correlation between measured β-lactamase activity and MIC, suggesting that non-β-lactamase-mediated resistance mechanisms play an important role in CFP resistance in *B. multivorans* LMG 18825. For *B. arboris* R-132 (Fig. 1b), the situation is different. We observed high β-lactamase activity (7.29) when cells were exposed to CAZ, and a high MIC for CAZ (128 mg/L). Combination treatment with SUL or AVI led to a statistically significant (*p* < 0.05, *n* = 4) decrease in β-lactamase activity (1.10 and 3.30, respectively), associated with an 8-fold MIC decrease for CAZ. These data suggest that for this Bcc strain β-lactamase is the major cause of resistance to CAZ. For *B. cenocepacia* LMG 16656 (Fig. 1c), no differences in MIC-values for CAZ (MIC = 512 mg/L) or CFP (MIC = 32 mg/L) were observed upon addition of a β-lactamase inhibitor (SUL, AVI or TAZ). β-lactamase activity assay results showed that treatment with CAZ or CFP induces β-lactamase activity in this strain, and that addition of a β-lactamase inhibitor to CAZ- or CFP-treatment has no significant (*p* > 0.05, *n* = 3) influence on β-lactamase activity. A likely explanation for this is that 15 of the 21 β-lactamases currently identified in the genome of *B. cenocepacia* LMG 16656 belong to the metallo-β-lactamase protein family [26]. As mentioned, this class of β-lactamases is not inhibited by any of the currently available β-lactamase inhibitors. The MIC- and β-lactamase activity results for *B. cenocepacia* LMG 16656 confirm that SUL, AVI or TAZ are not able to effectively inhibit metallo-β-lactamases and thus have no effect on the resistance of this strain against β-lactam antibiotics. Susceptibility of *B. vietnamiensis* LMG 18835 (Fig. 1d) for CAZ, CFP and AZT is altered upon addition of SUL; addition of SUL decreased the MIC for CAZ from 4 mg/L to 0.25 mg/L, for CFP from 16 mg/L to 1 mg/L and for AZT from 8 mg/L to 1 mg/L. However, relative β-lactamase activity for all three β-lactams is significantly increased upon addition of SUL. We currently have no explanation for these at first sight contradictory results, but it seems likely that both β-lactamase dependent and independent mechanisms are involved in β-lactam resistance in this strain. Results for the other Bcc strains investigated (see Additional file 2) confirm that for most Bcc strains investigated the influence of a β-lactamase inhibitor on β-lactamase activity is both strain- and/or antibiotic-dependent.

## Conclusions

There are considerable differences in susceptibility of Bcc strains to different β-lactam antibiotics. In the present study we investigated the effect of β-lactamase inhibitors on the susceptibility of Bcc species against β-lactam antibiotics. CLA had no effect on this susceptibility for any of the strains tested, but addition of SUL, TAZ or AVI to CAZ, AMOX, CFX, CFP or AZT leads to increased susceptibility (at least 4-fold decrease in MIC) in several Bcc strains. An important fact to take into account is that up to date, there is no clear evidence that in vitro susceptibility of Bcc species against a specific antimicrobial compound is related to clinical outcome [27].

Investigation of the relation between altered susceptibility upon addition of SUL, TAZ or AVI and differences in β-lactamase activity was performed with a β-lactamase activity assay kit. Pronounced differences in β-lactamase activity after exposure to different β-lactam antibiotics and β-lactamase inhibitors, as well as between the Bcc strains tested were observed. Some of the results observed are in line with our expectations, confirming that resistance against β-lactam antibiotics is mediated by expression of β-lactamase enzymes which can be successfully inhibited in vitro by β-lactamase inhibitors. However, results obtained with other strains suggest that non-β-lactamase-mediated resistance mechanisms to β-lactam antibiotics (likely including reduced membrane permeability, altered PBPs and presence of efflux pumps) are also important.

Production of β-lactamases is clearly not the only mechanism Bcc strains use to survive treatment with β-lactam antibiotics, and it is therefore questionable that adding β-lactamase inhibitors to β-lactam therapy will be a valuable approach to combat Bcc infections.

## Additional files

Additional file 1: Biological and geographic origin of all *Burkholderia cepacia* complex strains used in this study. (PDF 419 kb)
Additional file 2: β-lactamase activity and corresponding MIC-values for the complete selection of Bcc species studied in this work. Figures S1 – S5 show the β-lactamase activity and corresponding MIC-values for the remaining Bcc species which are not shown in the body of this manuscript. (PDF 1530 kb)

## Abbreviations

AMOX: Amoxicillin
AVI: Avibactam
AZT: Aztreonam
Bcc: *Burkholderia cepacia* complex
CAZ: Ceftazidime
CF: Cystic fibrosis
CFP: Cefepime
CFX: Cefoxitin
CLA: Clavulanic acid
EDTA: Ethylenediaminetetraacetic acid
EUCAST: European Committee on Antimicrobial Susceptibility Testing
LB: Luria Bertani
MBL: Metallo-β-lactamase
MEM: Meropenem
MHB: Mueller-Hinton broth
MIC: Minimal inhibitory concentration
PBPs: Penicillin binding proteins
SUL: Sulbactam
TAZ: Tazobactam
