# Supplementary material for: Effect of β-Lactamase inhibitors on in vitro activity of β-Lactam antibiotics against Burkholderia cepacia complex species
Source: Antimicrob Resist Infect Control. 2016 Nov 16;5:44. doi: 10.1186/s13756-016-0142-3 (PMC5111247; doi:10.1186/s13756-016-0142-3)
Supplement: Additional file 1: — Biological and geographic origin of all Burkholderia cepacia complex strains used in this study. (PDF 419 kb) [file 13756_2016_142_MOESM1_ESM.pdf]

Table S1. Biological and geographic origin of all *Burkholderia cepacia* complex strains used in this study.[1, 2]

Species	Strain	Biological origin	Geographic origin
B. cepacia	LMG 1222^{T}	Allium cepa	USA
	LMG 18821	CF patient	Australia
B. multivorans	LMG 18822	CF patient	Canada
	LMG 18825	CF patient	UK
	LMG 13010^{T}	CF patient	Belgium
	LMG 17588	Soil	Berkeley, USA
B. cenocepacia	LMG 16656^{T}	CF patient	Edinburgh, UK
	LMG 18828	CF patient	Canada
	LMG 18829	CF patient	USA
	LMG 18830	CF patient	Australia
B. vietnamiensis	LMG 10929^{T}	Oryza sativa, rhizosphere soil	Vietnam
	LMG 18835	CF patient	USA
B. ambifaria	LMG 19182^{T}	Pea rhizosphere	Wisconsin, USA
	LMG 19467	CF patient	Australia
B. lata	LMG 6992	Soil	Trinidad & Tobago
	R-9940	CF patient	Canada
B. stabilis	LMG 14294^{T}	CF patient	Belgium
	LMG 14086	Respirator	UK
B. dolosa	LMG 18943^{T}	CF patient	USA
	LMG 18941	CF patient	USA
B. anthina	LMG 20980^{T}	Soil rhizosphere	Nashville, USA
	LMG 20983	CF patient, sputum	Blackpool, UK
B. pyrrocinia	LMG 21824	CF patient	USA
B. ubonensis	LMG 20358^{T}	Surface soil	Thailand
	LMG 24263	Nosocomial infection	Thailand
B. latens	LMG 24064^{T}	CF patient	Italy
B. arboris	LMG 24066	Soil	USA
	R-132	CF patient	USA
B. seminalis	LMG 24067^{T}	CF patient	USA
	LMG 24272	Nosocomial infection	Thailand
B. metallica	LMG 24068^{T}	CF patient	USA
	R-2712	CF patient	Canada
B. contaminans	LMG 16227	CF patient, respiratory tract	Sweden
	R-12710	Sheep with mastitis, milk	Spain

'T' = Type strain

1. **BCCM/LMG Bacteria Catalogue** [http://bccm.belspo.be/catalogues/lmg-catalogue-search]
2. Mahenthiralingam E, Coenye T, Chung JW, Speert DP, Govan JR, Taylor P, Vandamme P: **Diagnostically and experimentally useful panel of strains from the Burkholderia cepacia complex.** *J Clin Microbiol* 2000, **38**:910-913.
