# Supplementary material for: Effect of β-Lactamase inhibitors on in vitro activity of β-Lactam antibiotics against Burkholderia cepacia complex species
Source: Antimicrob Resist Infect Control. 2016 Nov 16;5:44. doi: 10.1186/s13756-016-0142-3 (PMC5111247; doi:10.1186/s13756-016-0142-3)
Supplement: Additional file 2: — β-lactamase activity and corresponding MIC-values for the complete selection of Bcc species studied in this work. Figures S1 – S5 show the β-lactamase activity and corresponding MIC-values for the remaining Bcc species which are not shown in the body of this manuscript. (PDF 1530 kb) [file 13756_2016_142_MOESM2_ESM.pdf]

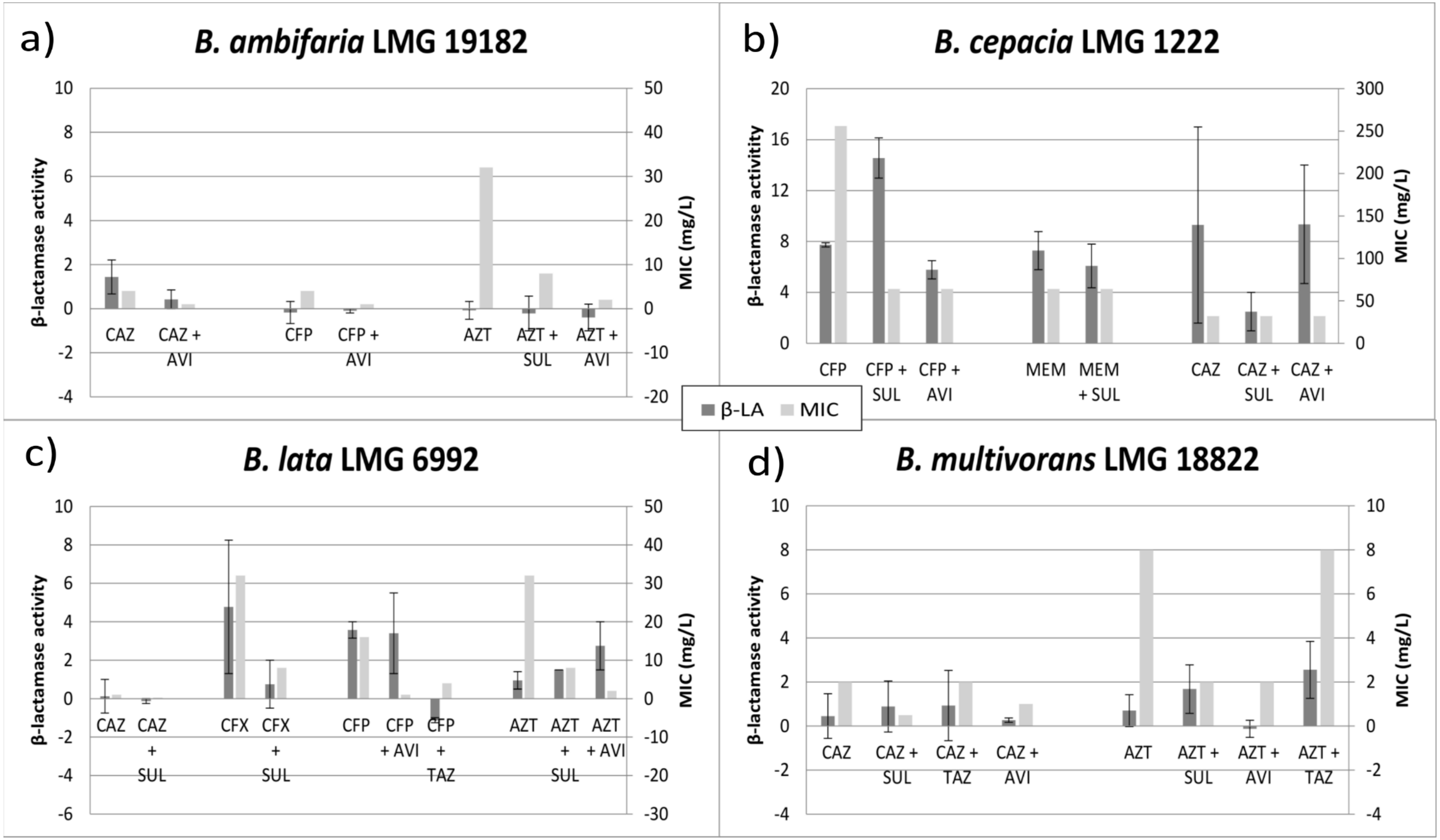


*Figure S1.* β-lactamase activity (dark grey bars, relative to untreated controls) and corresponding MIC values (light grey bars, mg/L) for a) *B. ambifaria* LMG 19182, b) *B. cepacia* LMG 1222, c) *B. lata* LMG 6992 and d) *B. multivorans* LMG 18822. Error bars represent min-max (n=2).

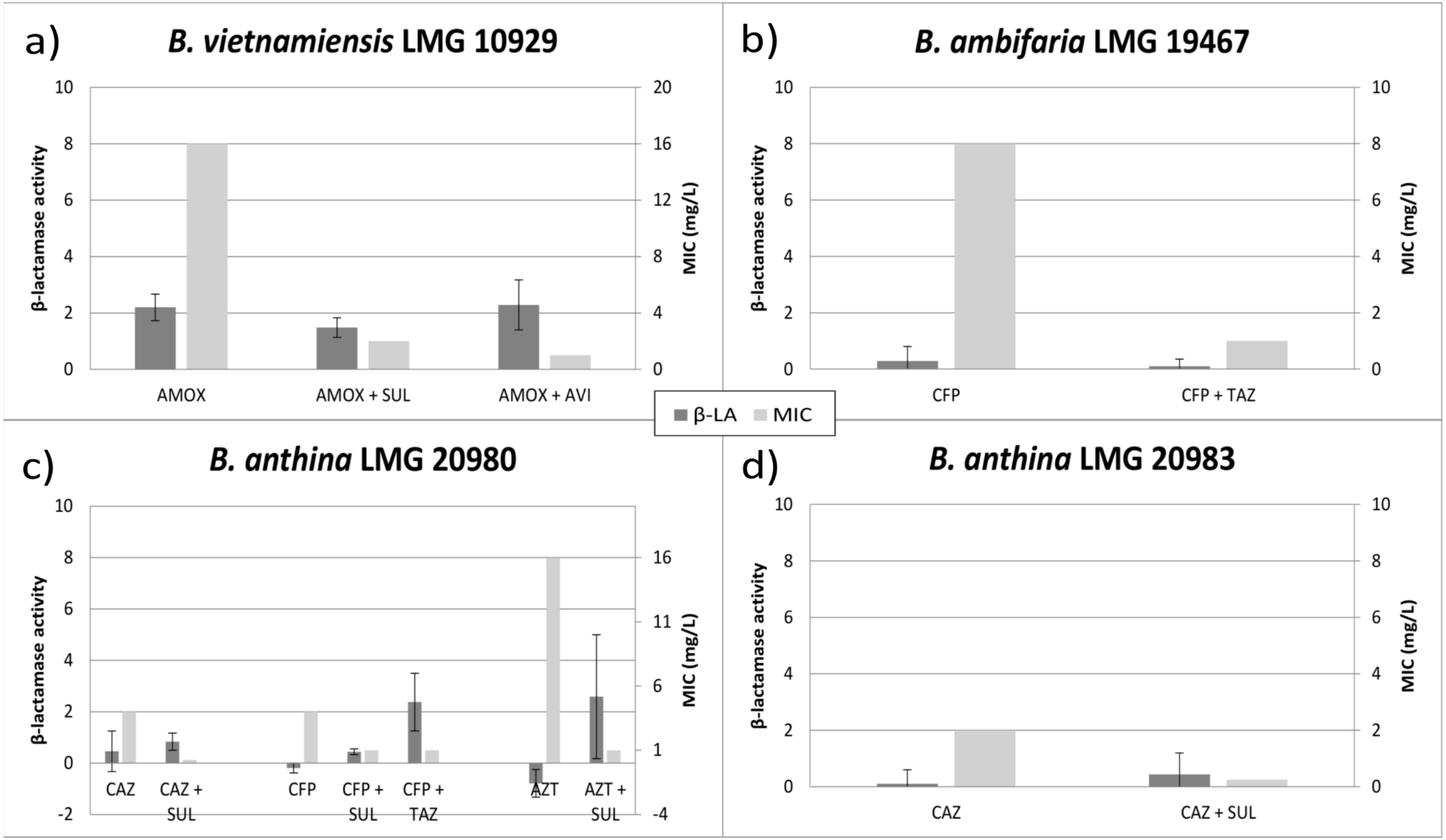


*Figure S2.* β-lactamase activity (dark grey bars, relative to untreated controls) and corresponding MIC values (light grey bars, mg/L) for a) *B. vietnamiensis* LMG 10929, b) *B. ambifaria* LMG 19467, c) *B. anthina* LMG 20980 and d) *B. anthina* LMG 20983. Error bars represent min-max (n=2).

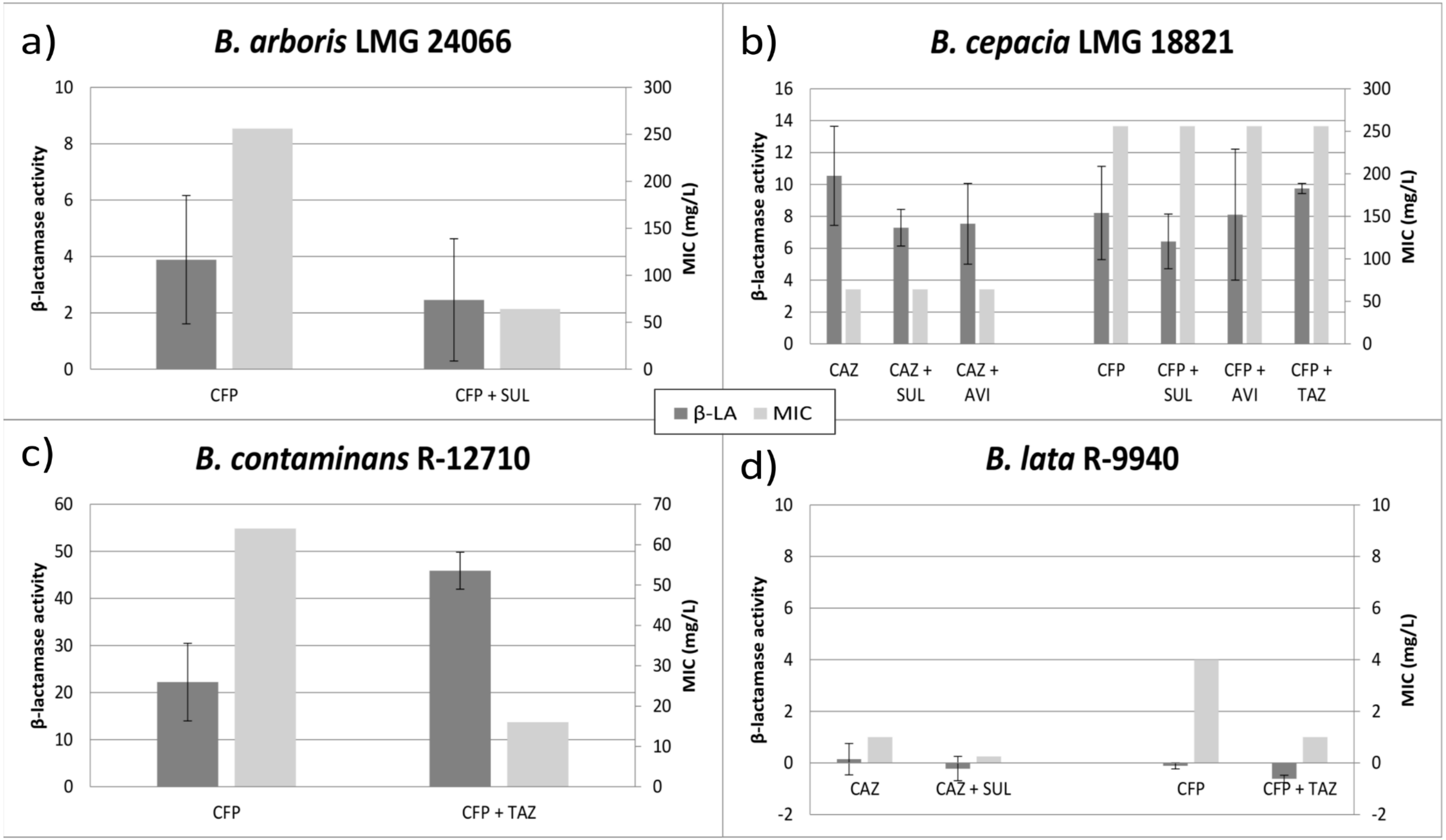


*Figure S3.* β-lactamase activity (dark grey bars, relative to untreated controls) and corresponding MIC values (light grey bars, mg/L) for a) *B. arboris* LMG 24066, b) *B. cepacia* LMG 18821, c) *B. contaminans* R-12710 and d) *B. lata* R-9940. Error bars represent min-max (n=2).

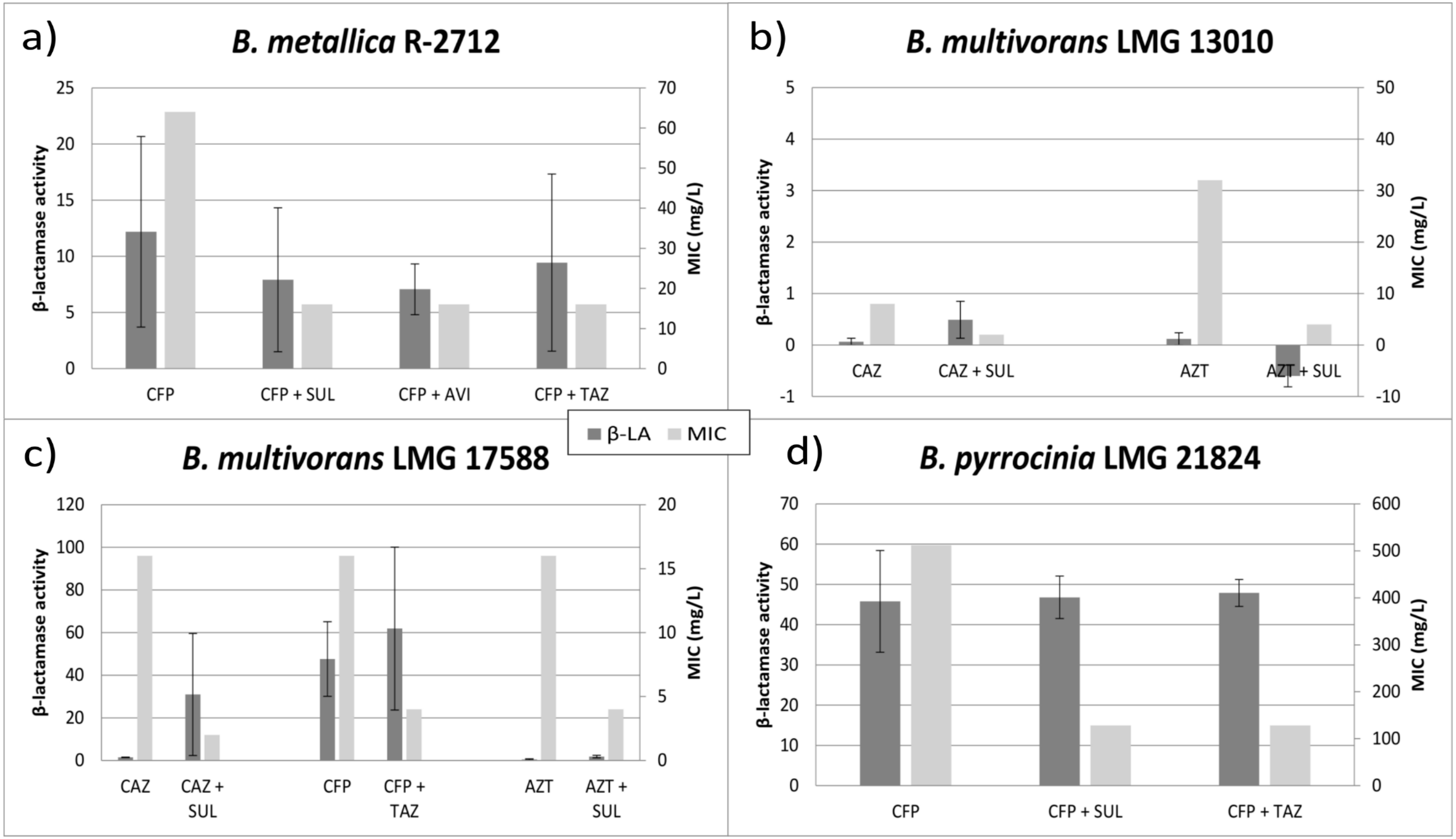


*Figure S4.* β-lactamase activity (dark grey bars, relative to untreated controls) and corresponding MIC values (light grey bars, mg/L) for a) *B. metallica* R-2712, b) *B. multivorans* LMG 13010, c) *B. multivorans* LMG 17588 and d) *B. pyrrocinia* LMG 21824. Error bars represent min-max (n=2).

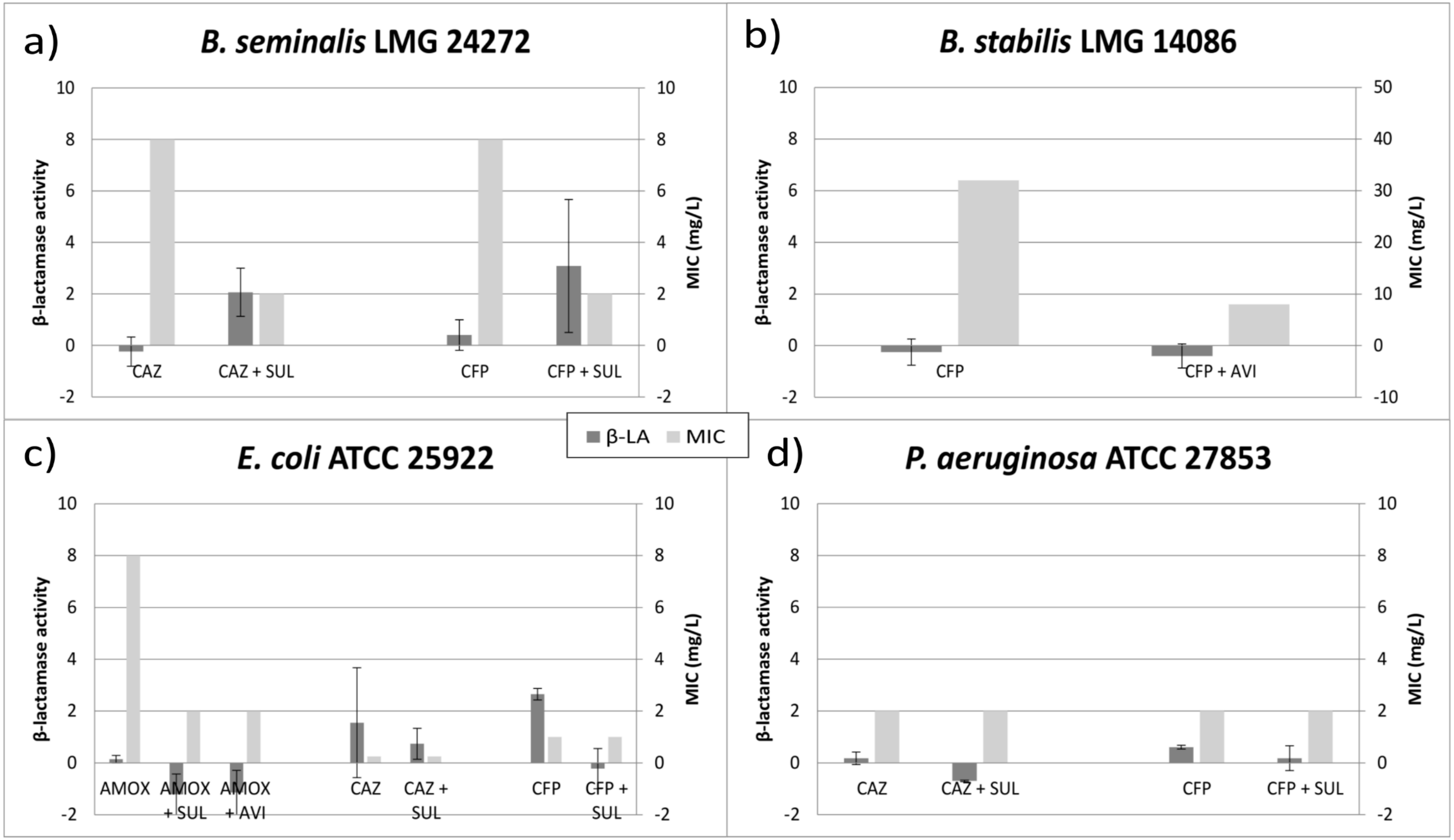


*Figure S5.* β-lactamase activity (dark grey bars, relative to untreated controls) and corresponding MIC values (light grey bars, mg/L) for a) *B. seminalis* LMG 24272, b) *B. stabilis* LMG 14086 and two control strains: c) *E. coli* ATCC 25922 and d) *P. aeruginosa* ATCC 27853. Error bars represent min-max (n=2).
